# Shrinking the language accessibility gap: a mixed methods evaluation of telephone interpretation services in a large, diverse urban health care system

**DOI:** 10.1186/s12939-015-0212-9

**Published:** 2015-09-15

**Authors:** Tatiana Dowbor, Suzanne Zerger, Cheryl Pedersen, Kimberly Devotta, Rachel Solomon, Kendyl Dobbin, Patricia O’Campo

**Affiliations:** LiKaShing Knowledge Institute, Centre for Research on Inner City Health, 209 Victoria Street, Toronto, Ontario M5C 1 N8 Canada; Health Integration Network of Toronto Central, 425 Bloor Street East, Toronto, Ontario M4X 1 L7 Canada; Dalla Lana School of Public Health, University of Toronto, Health Sciences Building, 6th floor, 155 College Street, Toronto, Ontario M5T 3 M7 Canada

## Abstract

**Introduction:**

Language interpretation services for patients who are not proficient in a country’s official language(s) are essential for improving health equity across diverse populations, and achieving clinical safety and quality for both patients and providers. Nevertheless, overall use of these services remains low, regardless of how they are delivered. In Toronto, Ontario, one of the most ethnically diverse urban centres, the regional local health integration network which oversees the highest concentration of health care organizations servicing 1.2 million residents, partnered with key stakeholders to make Over-the-Phone (OPI) interpretation services broadly and economically available in 170 different languages to its diverse network of health care organizations. This evaluation aimed to assess patients’ and providers’ experiences with OPI in these varied settings and the impact (if any) on alternative interpretation services and on health service delivery access and quality.

**Methods:**

This study used a two-phased sequential exploratory mixed-methods approach to evaluate the initiative. Phase I was comprised of semi-structured interviews with representatives from the program stakeholders; these findings were applied to identify appropriate survey questions and response categories, and provided context and depth of understanding to Phase II results. Phase II included web-based and self-administered surveys for both providers and patients engaging with OPI.

**Results:**

Both providers and patients identified a broad range of positive impacts OPI had on health care service delivery quality and access, and high levels of satisfaction with OPI, in a variety of health care settings. Providers also revealed a marked decrease in the use of ad-hoc, nonprofessional strategies for interpretation after the implementation of OPI, and noted it had either no impact on their workload or had decreased it overall.

**Conclusions:**

OPI is clearly not the sole answer to the complex array of health care needs and access gaps that exist for persons without proficiency in their country’s official language. Nevertheless, this evaluation provides compelling evidence that OPI is a valuable component, and that it may contribute to a broader range of positive impacts, and within a broader range of health care settings, than previously explored.

Language interpretation services to aid health care patients who are not proficient in a country’s official language(s) are essential to improve health equity across diverse populations, and achieve clinical safety and quality for both patients and providers [1–4]. The presence of such services has shown to improve patients’ and providers’ perceptions of higher quality of care, and general appreciation and satisfaction in a wide variety of settings [5–11]. They also increase access to primary care, including preventive services [12, 13].

Despite evidence of their value and importance, overall use of professional interpretation services remains low, regardless of how they are delivered. Studies have shown usage is low in Australia, for example, where interpreter services are more broadly available compared to other countries [14]. One review found just 18 % of hospitals in the United Kingdom used a formal interpreter agency [15]; and a review of pediatricians found low use in all states in the United States, regardless of demographics – a pattern which has improved only modestly since 2004 [16, 17]. The most commonly cited reasons for low uptake of these services include cost issues - such as lack of reimbursement mechanisms - and timeliness of access [18, 2, 19, 20, 6, 21, 17, 22]. Other noted barriers include presence of bilingual staff [23] and providers’ belief that patients prefer using their relatives over professional interpreters [24, 14], though some studies with patients have disputed the latter [5, 12].

## Telephone Interpretation Services (OPI)

Even in health care practices where interpretation services are widely available and encouraged by regional policies, telephone interpretation (hereafter OPI, for ‘over the phone interpretation’) tends to be unused and/or substituted by ad hoc non-professional methods of interpretation. Again, reasons for this vary, including an attachment to current practices and a general lack of awareness of its availability [2, 15, 24, 22]. OPI offers an interesting mix of benefits and challenges. Its impersonal quality enables confidential communication about sensitive or emotionally disturbing information [25, 20], but the loss of nonverbal input can also be a barrier to an optimal exchange [2, 26, 27, 6, 25, 28]. Providers also cite inconvenience and the extra time it requires as disadvantages of OPI [23, 26], and suggest its utility may be limited to certain types of provider-patient interactions, such as direct information exchange [29, 20].

The most common fall-backs, though, are problematic at best: reliance on patients’ family members and friends, or untrained bilingual volunteers or staff, as ad hoc interpreters should not be considered either satisfactory or sufficient. These ad hoc solutions are associated with impaired quality of care, frequent medical errors, and breaches of confidentiality [11, 10]. Indeed, a recent review found general consensus in the literature on this point, that language access services within health care should be considered “an essential component” to improve quality of care and reduce health disparities [3].

## Key gaps in the literature

Extant literature on OPI in health care settings, while ample, leaves many unanswered questions. Most of the research, for example, assesses experiences with and implementation of OPI in primary care settings, hospitals or shared networks of hospitals - mostly US-based; little is known about how OPI is experienced or functions within a more diverse network of health care organizations. Most OPI experiences focus on physician (and medical resident) usage patterns, and/or on professional interpreters. This means we have a limited understanding of how other allied health care professionals and patients within community health care settings respond to OPI and the effects it has on the use of other interpretation services.

## Toronto’s context and OPI model of service delivery

**Fig. 1:**
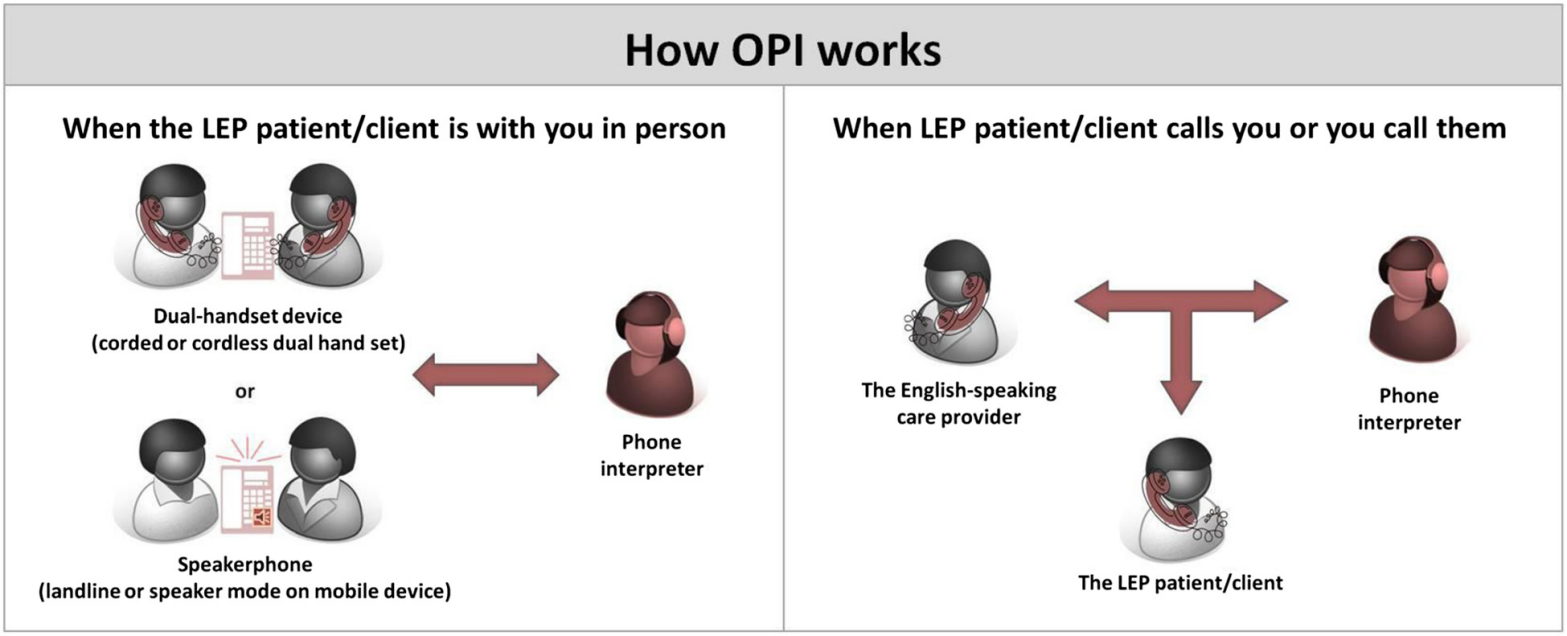
How OPI works. Graphic representation of the process for Over-the-Telephone Interpretation

Toronto, Canada, is one of the most ethnically diverse urban centres. According to the 2006 Census, 46 % of the metropolitan area population are immigrants, with approximately 170 languages and dialects represented within its population. This creates special concerns and challenges about the gaps in healthcare access [30], especially because no guidelines exist to ensure or monitor language interpretation services. In Ontario, 14 regional Local Health Integration Networks (LHINs) plan, integrate and fund local health care services; the Toronto Central LHIN (TC LHIN) is the largest of these, overseeing the highest concentration of health care organizations serving 1.2 million residents in its catchment area. When quality medical interpretation services emerged as a top health equity issue, the TC LHIN partnered with key stakeholders to make OPI services more broadly and economically available. In 2012, TC LHIN and partners made coordinated bulk-purchased real-time OPI services available in 170 different languages, 24 h a day, for its health care organizations. All interpreters completed extensive training, including medical interpretation and all aspects highlighted in the National Standard Guide for Community Interpretation Services [31]. Coordinated feedback from providers was also incorporated in the program as a quality control measure. Using a dual-handset device, speakerphone or teleconferencing telephone feature, patients with limited English can communicate with providers and other health care staff in their preferred language (refer to Fig. 1). Services are accessed through one central telephone number. Callers are prompted to key in the needed language and are transferred directly to an interpreter; if unavailable, they are transferred to a larger service to be connected with an interpreter. In the case of rare languages, and/or to ensure availability, staff can call ahead to pre-book an interpreter.

Each health care organization is provided an access code upon enrollment. Providers use the code to access services and track organization usage and direct billing. The TC LHIN covers the cost for health service providers in community health centres and other community-based support programs within its catchment area. Participating hospitals pay for the services, and benefit from the group rate and program coordination the TC LHIN provides. A more specific description of the model has been published elsewhere. [32, 33]

## Research questions

This evaluation assesses the initiative of the TC LHIN to provide expanded OPI services to a wide range of health care organizations, including community health centres and mental health and addictions centres, within its large downtown catchment area. Specifically, this mixed-methods study addresses gaps in the literature on OPI by addressing these primary questions:

- What are patients’ and providers’ experiences with OPI in these varied settings?
- What is the impact of OPI on alternative interpretation services and on service delivery access and quality? And,
- What are the most effective and appropriate uses of OPI in a shared network of diverse health care organizations?

### Methods

This study used a two-phased sequential exploratory mixed-methods approach to address the research questions [34]. All data were collected after the implementation of OPI. In Phase I, open-ended qualitative interviews were conducted with program stakeholders to inform the development of closed-ended surveys and provide context for overall study findings. These surveys were subsequently completed by healthcare providers and patients in Phase II of the study, making it possible to generalize the findings from phase I.

### Data sources

#### I: Qualitative phase

Purposive sampling was used to select organizations with a variety of program usage patterns (low, medium and high), and of different sizes (small and large) and types (e.g., hospital, community health centre). All selected sites (*n* = 9) had at least one provider and one manager participate in the qualitative phase. The refusal rate during recruitment was 10 %. Semi-structured interviews were conducted with 31 program stakeholders who had experience with OPI; they included two members of the TC LHIN leadership, 10 service managers, 17 service providers, and two administrative staff. The four interview guides, one for each type of stakeholder,focused on experiences with the OPI program from introduction through implementation.

#### II: Descriptive cross-sectional quantitative phase

Surveys for providers and patients were developed based on themes identified in the literature as well as those uncovered in Phase I. All surveys were web-based and self-administered. Both providers and patients were provided with web links to complete the survey, though patients were also given the option of completing a paper survey that they returned to the research team using a postage-paid envelope. Surveys were distributed to all 34 sites that had signed on for and had used the OPI program at the time of the evaluation. The organizations’ managers recruited service providers to complete the provider survey, and service providers recruited patients to complete the patient survey. This convenience sampling approach resulted in a sample of 127 providers (representing 30 of the 34 sites, or 88 %) and 41 patients. The provider survey contained closed- and open-ended questions about changes in communication strategies, frequency of usage, impact on patients, care appropriateness, and program satisfaction. The closed-ended patient survey asked about their satisfaction with the interpretation services, opinions about its impacts, and what they would do if it were not available. The patient survey was translated into the top 10 languages accessed through the OPI program at the time of the evaluation.

### Ethics review

The St. Michael’s Hospital Research Ethics Board deemed this study a quality improvement initiative which did not require a comprehensive Board review. Criteria for this decision are outlined in the *Tri-Council Policy Statement: Ethical Conduct of Research Involving Humans, 2nd edition*.

### Data analysis

#### I: Qualitative phase

Interviews were audio-recorded and transcribed verbatim by a professional transcription service. An initial sample of the transcriptions was coded by three members of the research team to develop a codebook; once they reached consensus on discrepancies, one member coded the remaining interviews. The team used NVivo 9.0 software to assist in the coding and store memos. Thematic analysis was applied to identify appropriate survey questions and accompanying response categories. For example, discussions of the most commonly experienced barriers and issues faced with accessing interpretation services were reviewed to craft relevant survey questions to capture frequency among the larger group of survey respondents. These interviews also provided context and depth of understanding for survey results.

#### II: Quantitative phase

Snap 11 survey software was used to program both the patient and provider surveys, and to store the data. The data were exported from Snap into Microsoft Excel, which was used to calculate descriptive statistics.

### Results

#### Characteristics of respondents

##### Providers

One-hundred and twenty-seven providers completed surveys, representing 88 % (30 of 34) of participating sites. All health care sectors were represented, with 40 % from hospitals, 37 % from community health centres, and 23 % from community support services, including community mental health and addictions. The largest groups of providers were nurses and social workers, representing 22 and 16 % of the sample respectively. Others included doctors, dietitians, case managers, care coordinators, technologists, administrative staff, and management. Eighty-five percent were female, and 84 % fell within the age range of 25 to 54 (39 % were 25–34 years).

##### Patients

Forty-one patients completed the survey, representing 68 % (23 of 34) of participating sites. Patients reported about all sites where they had used OPI; 61 % had accessed OPI at community health centres, 59 % at a hospital and 32 % at community support services. The majority (78 %) of respondents were female. Forty percent were 25–44 years old, and one-quarter 65 years and older. Most participants identified as White/European (32 %), East Asian (26 %) and Latin American (16 %). The top languages preferred by survey respondents were Portuguese (22 %) and Spanish (20 %); the next most represented languages were Russian, Cantonese, Vietnamese and Korean.

#### Effects on use of alternative interpretation sources prior to and after OPI

Half (52 %) of the providers reported ‘often’ or ‘always’ relying on assistance of family and friends to interpret prior to the implementation of OPI, and an additional 37 % reported using them at least some of the time. Nearly one-quarter (23 %) used untrained administrative staff for interpretation help ‘often’ or ‘always’, and over one-third (35 %) relied on other providers (refer to Table 1). One-tenth (11 %) requested patients to bring their own interpreters to appointments, and 6 % relied on other patients to help with interpretation ‘often’ or ‘always.’

**Table 1:** Strategies used “Often or Always” prior to and after OPI implementation

*(5-point likert scale responses: never, rarely, sometimes, often, always)*		
(*n* = 127)*		
	*% reporting strategies used before implementation of OPI*	*% reporting strategies used after implementation of OPI*
Assistance of patient’s family and/or friends who speak needed language	52	No data available
Face-to-face professional interpreters	37	24
Assistance of other providers who speak needed language	35	16
Assistance of administrative staff who speak needed language	23	11
Asking patients to bring their own interpreters to appointments	11	4
Assistance of other patients who speak needed language	6	7
Volunteer language interpreters	4	2
Referrals to other agencies	3	2

^*^Percentages are based on valid responses only

Interview respondents described several ethical and practical concerns with relying on family or staff. Issues related to family members, for example, included lack of training (“you don’t know what they’re translating; they’re not bound by any sort of ethical training”) and sensitive situations for which they do not want to involve family or friends. Similarly, health care providers worried about non-clinical staff’s capability (“regular communication can be quite different than communication pertaining to medical issues”) as well as taking them away from their own work (“when you’re pulling other nurses to come and translate for you, when you’re pulling housekeeping… you’re pulling them away from their work.”)

When asked to compare their interpretation practices prior to and after OPI implementation, these providers revealed a marked decrease in the use of ad-hoc, non-professional strategies. Reliance on other providers or administrative staff, for example, decreased by about half, and requesting patients to bring their own interpreters decreased even more substantially. The use of face-to-face professional interpreters also decreased considerably, from 37 to 24 %, primarily due to conveniences associated with OPI such as not needing to book ahead of time.

#### Effects on health care quality and access

Providers and patients expressed similar perspectives on the overall quality of care with OPI (84 % of providers; and 85 % of patients reported it was ‘improved’ or ‘significantly improved’), and on several aspects of patient engagement, including overall comfort level (both, 72 %), relationships between providers/patients (72 %; 68 %), and disclosure of patients (68 %; 72 %)(refer to Table 2). On the whole, about three-quarters or more of both groups reported overall improvements on all of the measures of patient engagement with their provider and their care. One exception was just half of patients saw improvements in their privacy after OPI was available, compared with two-thirds of providers who did, though this difference was not statistically significant.

**Table 2:** Effects of OPI on health care quality and patient engagement: perspectives of providers and patients

*(5-point likert scale responses: significantly decreased, decreased, neither increased or decreased, increased, significantly increased)*		
	Providers (n = 127)*	*Patients (n = 41)*
*How has the use of the OPI affected the following aspects of health care provision for patients who used the program?*	*Percent reporting “Improved” or “Significantly Improved”*	
Overall quality		
Overall quality of care	84	85
Patient engagement with Providers/Care		
Patient’s comfort level	72	72
Relationship between provider and patient	71	68
The disclosure of patients	68	72
Patient’s privacy	67	51
Patient autonomy	78	-
Patient engagement	78	-
Patient access to you organization	73	-
Understanding of information given during appointment	-	87
Likelihood to ask questions during the visit	-	84
Likelihood to recommend the health care organization to other friends and family who speak the same language	-	82
Ability to schedule follow-up or future appointments on time	-	75
Ability to follow health care provider’s instructions	-	74

*Percentages are based on valid responses only, which ranged from 120–127

Questions asked only of patients reveal additional aspects of ‘quality’ and ‘access’; for example, four-fifths or more said they had a greater understanding of information given them during their appointment (87 %) and were more likely to ask questions (84 %); three-quarters reported an increased ability to follow through on providers’ instructions and on scheduling follow-up appointments. A majority (82 %) also said they would recommend the organization to other family members and friends as a result of its OPI capability.

In qualitative interviews, respondents frequently described these issues – access, quality, comfort, engagement, and autonomy - as closely interrelated. One health care provider, for example, called OPI a “wonderful service, which has increased our access and timeliness in reaching out to our clients in a manner which makes them most comfortable and able to participate in the arrangement of their care.” Another noted, “Interpretation is really important for maintaining the health of the individuals in the community. It is very important they understand instructions really well, and that they express what they want to tell physicians really well to be able to get good health care.”

A large majority of the providers considered OPI ‘appropriate’ for encounters involving supportive (90 %), acute (88 %), and chronic (86 %) care. They raised more concerns about its appropriateness for mental health encounters; noting, for example, the special importance of building rapport and trust, and a need to sensitively interact with patients. Nonetheless, most rated it either ‘appropriate’ (73 %) or ‘somewhat appropriate’ (19 %) in mental health encounters.

Asked to speculate about how the loss of OPI might affect themselves and their organization, 81 % providers said they would struggle to engage with patients, nearly three-quarters noted the quality (74 %) and efficiency (71 %) of care would decrease, and nearly two-thirds reported patients’ access to care (64 %) would be compromised (refer to Table 3). Half (49 %) of the patients said they would revert to asking friends or family members to help them, and one-fifth (17 %) said they would try to interpret on their own and another one-fifth (17 %) indicated they did not know what they would do. One-third (32 %) of patients said it would mean seeking another health care provider.

Health care providers described these impacts in strong language (“a lifeline that is cut, because communication is everything for us”) and warned some patients are especially isolated and would lose access entirely. As one provider commented, for example: “a few clients…don’t have anyone who could translate for them and they don’t speak English, and for them I don’t know what we would do. They wouldn’t be able to access care.”

**Table 3:** Perceived impact of loss of OPI

*If the OP services were cut, what would be the impact to your organization?*	*% of providers (n = 127)*
Increased difficulty for staff to engage patients	81
Decreased quality of care	74
Decreased efficiency of care (time)	71
Decreased use of phone interpretation	68
Patient access to care would be compromised	64
Increased financial cost to offer interpretation	60
Impact on reputation of organization (organization would no longer be seen as accessible to non-English speaking patients)	44
*What would you do if the OPI services are not offered anymore by the organization where you received it?*	*% of Patients (n = 41)*
Ask a friend/family member to help me with interpretation	49
Find a health care provider who speaks my language	32
Stop going to the organization and find another one that offers interpretation	20
Try to understand what providers say without the help of interpreters	17
Don’t know	17

#### Satisfaction

A large majority of both providers (93 %) and patients (85 %) reported being ‘satisfied’ or ‘very satisfied’ with OPI services overall (refer to Table 4). Their satisfaction with various aspects of the services, including timeliness of access, and quality and professionalism of interpreters, was also very high and closely aligned. A large majority (more than 80 %) of patients expressed satisfaction with issues related to their communication and comfort with the interpreter and the health care provider. Approximately two-thirds of providers expressed satisfaction with program coordination, training, and materials; most of the remainder rated these as ‘neutral’ (neither satisfied nor dissatisfied) and dissatisfaction ratings were rare.

**Table 4:** Provider and patient satisfaction with OPI

*How satisfied are you with the following aspects of the over-the-phone interpretation?*	*Percent reporting “Satisfied” or “Very satisfied”*	
	*Providers (n = 127)*	*Patients (n = 41)*
Overall satisfaction	93	85
Timely access to interpreters/available in needed languages	92	83
Quality of interpretation	95	88
Professionalism of interpreters	92	83
Confidentiality of interpretation	91	-
Wait times	89	-
Technology and equipment availability	83	-
Training to use the program	72	-
Program coordination/management	70	-
Program reference materials	63	-
TC LHIN leadership	55	-
Relationship with doctor or health care provider	-	93
Confidence in interpretation	-	90
Your understanding of information provided during appointments	-	90
Your ability to communicate with the doctor or health care provider	-	90
Your comfort level during appointments	-	85
Quality of telephone equipment	-	76

### Discussion

This mixed methods study assessed the experiences and perceived impacts of making OPI accessible to a variety of hospitals, clinics, and community health care providers in Toronto, Ontario, a diverse urban centre. As noted previously, most of the research on OPI services has heretofore focused on experiences of physicians and medical residents within hospital settings. This obscures the reality that patients are accessing health care in a wide range of settings from providers of various disciplines. For example, while a majority (59 %) of our patient sample used interpreter services in a hospital setting, 93 % had also accessed interpreter services in a community health facility. This high usage suggests great potential to expand the reach of OPI into a broader range of settings where patients access services. One systematic review found cost to be the primary barrier to use of OPI in community healthcare settings; by removing this barrier for community-based health centres and support programs, this initiative has already seen strong uptake of OPI [20].

As we consider implementation of OPI into more community-based settings, we also need to heed the voices of a more diverse group of healthcare providers; our sample, for example, included nurses, social workers, case managers, and care coordinators. With rare exception in this study, provider and patient experiences with, and opinions about, OPI strongly converged. While the literature consistently shows patient satisfaction related to OPI, provider satisfaction tends to be mixed [9, 25]; the level of convergence we saw may be attributable in part to the diversity of providers included in our study and/or the absence of preferable alternatives in community-based settings. In general, this study suggests more research is needed to understand the interpretation needs and experiences of patients and providers in more diverse healthcare settings.

Our mixed methods approach and diverse participant sample also revealed a broad range of positive impacts that OPI has on health care service delivery; again, prior research has tended to focus on a relatively narrow set of outcomes (e.g., ease/cost of use, communication quality, satisfaction). This study found providers and patients reported improved patient-provider relationships, comfort and privacy, but also an increased capacity and likelihood to schedule follow-up appointments, follow health care providers' instructions, disclose information, ask questions, and recommend the health care organization to family and friends. Patients and providers largely agreed on this broad range of positive impacts associated with patient autonomy and health care accessibility. While this provides a general endorsement for OPI, it also suggests that a better understanding of the range of benefits it can provide would help to inform and customize the role that OPI plays for patients and providers in a variety of settings. It is also notable that providers’ interview responses revealed strong interconnectedness between these impacts, exposing a far more nuanced and multifaceted nature of general concepts like health care access and quality. Measures of quality, for example, should arguably encompass issues such as a patient’s comfort disclosing to a provider, understanding of information the provider offers, and a likelihood to ask questions during the visit.

A large majority (86–90 %) of these providers considered OPI appropriate for encounters involving supportive, acute and chronic care. This is a more expansive endorsement than found in the extant literature, which generally concludes that OPI is most acceptable for simple, brief appointments not requiring visual communication, such as administrative, ancillary, or follow-up meetings [20, 29]. And, while the providers in this study raised more concerns about OPI’s appropriateness for mental health encounters, most still deemed it appropriate (73 %) or somewhat appropriate (19 %). Asked to explain ratings other than ‘appropriate,’ most of the concerns related to a personal preference for face-to-face interactions, a need for better understanding of mental health symptoms, and the cumbersome nature of the technology. Much of the provider (again, primarily physicians) resistance discussed in the literature references a range of implementation problems, such as rooms not wired for telephone use, lack of interpreter training, and long wait times; however, misconceptions about its use, aspersions about its value based on past experiences, and/or contentment with the status quo are nearly as common [14, 21, 24). Indeed, one author concluded that a key factor which impedes research and “stymie(s) professional debate” on the effectiveness and impact of telephone interpretation is “the persistence of (usually unsubstantiated) myths and stereotypes of TI” [35]. The relatively greater openness of providers toward OPI in this study, all of whom had used it in practice, suggests it may have broader applicability than previously assumed, particularly when preferable alternatives are unavailable. It is also noteworthy that all but one of the providers reported, during qualitative interviews, that access to OPI had either no impact on their workload or had decreased it overall. In general, though, it is critical that reasons underlying resistance be exposed and better understood if we are to effectively address them.

Frequency of reliance on family and friends – though we know it is fraught with issues – was used pre-OPI at least some of the time by 90 % of these providers – 52 % used them often or all of the time; reliance on other ad hoc solutions such as other providers or administrative staff were also common. Asked what they would do should OPI no longer be available, 81 % of providers said they would struggle to engage with patients, and nearly as many noted overall quality and efficiency of care would decrease and/or that patients access to care would be compromised. Half of patients said they would revert to asking family/friends, and a strong minority reported their access to care would be negatively affected. This, and providers’ comments that some patients would lose access entirely, reinforces the importance of finding a coordinated solution to interpretation needs within all of these health care settings. Failing to offer access to quality language interpretation services is clearly incompatible with a commitment to health equity.

This sample had broad representation from the organizations, providers, and patients accessing OPI as part of this initiative, and the two-phase exploratory design enabled development of well-tailored survey instruments. This evaluation also had several limitations, including a lack of qualitative input from patients due to logistical barriers. The use of convenience sampling means it is possible that survey respondents differed from non-respondents on important characteristics, and the completion of surveys primarily occurred in non-acute health situations, which may also have affected results. Our strategy of asking the intervention group to rate their experiences before and after the OPI program implementation is also prone to recall bias. On the whole, however, these methodological decisions enabled us to obtain information from a larger variety of respondent types and healthcare settings than we otherwise could have; future research should expand on these promising findings.

### Conclusions

OPI has advantages and disadvantages, and is clearly not the sole answer to the complex array of health care needs and access gaps that exist for persons without English proficiency. Nevertheless, this evaluation provides compelling evidence that OPI is a valuable component, and that it may contribute to a broader range of positive impacts, and within a broader range of health care settings, than previously explored.

## Abbreviations

OPI: Over the Phone Interpretation
TC LHIN: Toronto Central Local Health Integration Network

## References

[1] Karliner LS, Jacobs EA, Hm Chen A, Mutha S (2007). Do professional interpreters improve clinical care for patients with limited English proficiency? A systematic review of the literature. Health Serv Res.

[2] Gadon M, Balch GI, Jacobs EA (2007). Caring for patients with limited English proficiency: The perspectives of small group practitioners. J Gen Intern Med.

[3] Alliance A (2009). Literature review: Costs of not providing interpretation in health care.

[4] Loignon C, Hudon C, Goulet E, Boyer S, De Laat M, Fournier N et al. Perceived barriers to healthcare for persons living in poverty in Quebec, Canada: the EQUIhealThY project. Int J Equity Health. 2015;14(4). doi:10.1186/s12939-015-0135-5. 10.1186/s12939-015-0135-5 PMC4300157 25596816

[5] Jones D, Gill P, Harrison R, Meakin R, Wallace P (2003). An exploratory study of language interpretation services provdied by videoconferencing. J Telemed Telecare.

[6] Chan Y-F, Algapan K, Rella J, Bentley S, Soto-Greene M, Martin M (2010). Interpreter services in emergency medicine. J Emerg Med.

[7] Krugman SD, Parra-Roide L, Hobson WL, Garfunkel LC, Serwint JR (2009). Spanish-speaking patients perceive high quality care in resident continuity practices: A CORNET study. Clin Pediatr.

[8] Azarmina P, Wallace P (2005). Remote interpretation in medical encounters: A systematic review. J Telemed Telecare.

[9] Flores G (2005). The impact of medical interpreter services on the quality of health care: A systematic review. Med Care Res Rev.

[10] Juckett G, Unger K (2014). Appropriate use of medical interpreters. Am Fam Physician.

[11] Langer T, Wirth S (2014). Overcoming language barriers with telephone interpreters: first experiences at a German children's hospital. Zeitschrift fur Evidenz Fortbuildung und Qualitat im Gesundheitswesen.

[12] Gerrish K, Chau R, Sobowale A, Birks E (2004). Bridging the language barrier: the use of itnerpreters in primary care nursing. Health Soc Care Community.

[13] Brach C, Fraser I, Paez K (2005). Crossing the language chasm: An in-depth analysis of what language-assistance programs look like in practice. Health Aff.

[14] Phillips C (2010). Using interpreters: A guide for GPs. Aust Fam Physician.

[15] Hammick M, Featherstone C, Benrud-Larson L (2001). Information giving procedures for patients having radiotherapy: A national perspective of practice in the United Kingdom. Radiography.

[16] Kuo DZ, O'Connor KG, Flores G, Mikovitz CS (2007). Pediatricians' use of language services for families wtih limited English proficiency. Pediatrics.

[17] DeCamp LR, Kuo D, Flores G, O'Connor KG, Minkovitz C (2013). Changes in language services use by US pediatricians. Pediatrics.

[18] Hornberger J, Itakura H, Wilson SR (1997). Bridging language and cultural barriers between physicians and patients. Public Health Rep.

[19] Rose DE, Tisnado DM, Malin JL, Tao ML, Maggard MA, Adams J (2010). Use of interpreters by physicians treating limited English proficient women with breast cancer: Results from the provider survey of the Los Angeles women's health study. Health Serv Res.

[20] Masland M, Lou C, Snowden L (2010). Use of communication technologies to cost-effectively increase the availability of interpretation services in healthcare settings. Telemed J E-Health.

[21] Kazzi GB, Cooper C (2003). Barriers to the use of interpreters in emergency room paediatric consultations. J Paediatr Child Health.

[22] Sandler R, Myers L, Springgate B (2014). Resident physicians' opinions and behaviors regarding the use of interpreters in New Orleans. South Med J.

[23] Atkin N (2008). Getting the message across: Professional interpreters in general practice. Aust Fam Physician.

[24] Huang Y-T, Phillips C (2009). Telephone interpreters in general practice: Bridging the barriers to their use. Aust Fam Physician.

[25] Hsieh E (2006). Understanding medical interpreters: Reconceptualizing bilingual health communication. Health Commun.

[26] Ramsey KW, Davis J, French G (2012). Perspectives of Chuukese patients and their health care providers on the use of different sources of interpreters. Hawaii J Med Public Health.

[27] Riddick S. Improving access for limited English-speaking consumers: A review of strategies in Health Care Settings. J Health Care Poor Underserved. 1998;9(Supplement).

[28] Zaw R, Faulkenberry-Miranda C, Zuniga S, Ortiz C, Stoltz G, Yang S (2013). Barriers to clear communication for pediatric primary care providers when using phone interpreters: A focus group study. J Invest Med.

[29] El P, Perez-Stable EJ, Nickleach D, Lopez M, Karliner LS (2012). Interpreter perspectives of in-person, telephonic, and videoconferencing medical interpretation in clinical encounters. Patient Educ Couns.

[30] Sears J, Khan K, Adern CI, Tamim H (2013). Potential for patient-physician language discordance in Ontario. BMC Health Serv Res.

[31] Toronto Central LHIN. Language Services Toronto: Information for Interested Participants. Toronto, Toronto Central LHIN. 2013.

[32] Goar C. Toronto’s diverse population requires multilingual health care: Commentary. Toronto Star. 2014

[33] Centre for Research on Inner City Health. Reducing the Language Accessibility Gap: Language Services Toronto Program Evaluation Report. Toronto: Toronto, CRICH Survey Research Unit; 2014.

[34] Creswell JW (2015). A Concise Introduction to Mixed Methods Research.

[35] Ozolins U (2011). Telephone interpreting: Understanding practice and identifying research needs. Int J Translat Interpretat Res.

